# Impact of Obstructive Sleep Apnea on Liver Fat Accumulation According to Sex and Visceral Obesity

**DOI:** 10.1371/journal.pone.0129513

**Published:** 2015-06-15

**Authors:** Yoshiro Toyama, Kiminobu Tanizawa, Takeshi Kubo, Yuichi Chihara, Yuka Harada, Kimihiko Murase, Masanori Azuma, Satoshi Hamada, Takefumi Hitomi, Tomohiro Handa, Toru Oga, Tsutomu Chiba, Michiaki Mishima, Kazuo Chin

**Affiliations:** 1 Department of Respiratory Medicine, Graduate School of Medicine, Kyoto University, Kyoto, Japan; 2 Department of Respiratory Care and Sleep Control Medicine, Graduate School of Medicine, Kyoto University, Kyoto, Japan; 3 Department of Diagnostic Imaging and Nuclear Medicine, Graduate School of Medicine, Kyoto University, Kyoto, Japan; 4 Department of Respiratory Medicine, Otsu Red Cross Hospital, Shiga, Japan; 5 Department of Respiratory Medicine, Kansai Electric Power Hospital, Osaka, Japan; 6 Department of Clinical Laboratory, Graduate School of Medicine, Kyoto University, Kyoto, Japan; 7 Department of Gastroenterology and Hepatology, Graduate School of Medicine, Kyoto University, Kyoto, Japan; National Institute for Viral Disease Control and Prevention, CDC, CHINA

## Abstract

**Rationale:**

Associations between obstructive sleep apnea (OSA) and liver fat accumulation have been frequently investigated because both morbidities are common. Visceral fat was reported to be closely related to OSA and liver fat accumulation. Recently, sex differences in the association between OSA and mortality have gained much attention.

**Objectives:**

To investigate the associations among OSA, liver fat accumulation as determined by computed tomography, and visceral fat area and their sex differences.

**Methods:**

Studied were 188 males and 62 females who consecutively underwent polysomnography and computed tomography.

**Results:**

Although the apnea-hypopnea index was positively correlated with liver fat accumulation in the total males, none of the OSA-related factors was independently associated with liver fat accumulation in either the total male or female participants in the multivariate analyses. When performing subanalyses using a specific definition for Japanese of obesity or visceral obesity (body mass index (BMI) ≥25 kg/m^2^ or visceral fat area ≥100 cm^2^), in only males without visceral obesity, percent sleep time with oxygen saturation <90%, in addition to BMI, insulin resistance, and serum triglyceride values, was independently correlated with liver fat accumulation (R^2^ = 15.1%, P<0.001). In males, percent sleep time of oxygen saturation <90% was also a determining factor for alanine aminotransferase values regardless of visceral fat area. In contrast, OSA was not associated with liver fat accumulation or alanine aminotransferase values in females whether or not visceral obesity was absent.

**Conclusions:**

Sex differences in the visceral fat-dependent impact of OSA on liver fat accumulation existed. Although the mechanisms are not known and ethnic differences may exist in addition to the specific criteria of visceral obesity in Japan, the treatment of male patients with OSA might be favorable from the viewpoint of preventing liver fat accumulation and liver dysfunction even in patients without obvious visceral fat accumulation.

## Introduction

Obstructive sleep apnea (OSA) is characterized by nocturnal intermittent hypoxemia represented as the number of episodes of oxygen desaturation per hour over total sleep time (oxygen desaturation index: ODI) and sleep-associated hypoxia represented as the percentage of time spent with oxygen saturation (SpO_2_) below 90% to total sleep time (%T<90). Fatty liver disease is the most common chronic liver disease worldwide [1]. Fatty liver disease is a progressive disease from simple steatosis to steatohepatitis, liver cirrhosis, and hepatocellular carcinoma. Thus, both OSA and fatty liver disease are highly prevalent and each has an impact on patients’ prognosis.

During the decade following the first study [2], OSA and fatty liver disease have been attracting interest as an important target of study, and many studies have shown that OSA is associated with the progression of fatty liver disease by histology, radiology, and biomarkers [3–12]. Among studies investigating the association histologically, Mishra et al., Polotsky et al., and Aron-Wisnewsky et al., respectively, reported the lowest SpO_2_ during sleep, a mean fall in SpO_2_ caused by OSA, and the ODI to be associated with inflammation and fibrosis in fatty liver [6, 8, 10]. These findings showed that hypoxia related to OSA should play an important role in the progression of fatty liver disease. However, it is still unclear whether OSA is associated with the onset or early stage of fatty liver disease observed as liver fat accumulation (LFA). In addition, although recent human studies have mainly investigated the association between OSA and fatty liver disease in morbidly obese participants [5–11], in animal models of OSA, exposure to intermittent hypoxia for 5 days increased liver triglyceride content in lean mice without fatty liver but not in obese mice with fatty liver already at baseline, and it took 12 weeks to identify a similar and significant increase in obese mice [13, 14]. In these studies, sterol regulatory element binding protein 1 (SREBP-1), a key transcription factor of lipid biosynthesis, and stearoyl-coenzyme A desaturase 1 (SCD-1), an SREBP-1-induced enzyme of lipid biosynthesis, were upregulated in the liver in parallel to the increase in liver triglyceride content. It took 5 days in the lean mice and 12 weeks in the obese mice until the activation of SREBP-1 became significant. However, the mechanisms of SREBP-1 and SCD-1 activation or the rationales for the differences in the duration of intermittent hypoxia exposure were not clearly reported [13, 14]. Thus, an association among the hypoxemia induced by OSA, LFA, and obesity (especially the visceral fat accumulation **(**VFA), which is more influential in LFA than the body mass index (BMI) [1, 15]) has not been established. In addition, the thresholds of the degree of obesity or the VFA required for a significant association between OSA and LFA are not well known.

On the other hand, sex differences have been recognized in the epidemiology of fatty liver disease, such as its prevalence, severity, and prognosis [1, 16], and sex hormones and differences in patterns of body fat accumulation, including VFA, are considered to be main etiologies of sex differences [1, 17]. Thus, sex must be considered an important factor in fatty liver disease. Nevertheless, a direct comparison of OSA and fatty liver disease between males and females has not been made by radiological studies.

From this background, it has been suggested that it is important to understand the association among sex, VFA, OSA, and OSA-induced hypoxemia. We hypothesized that there is a sex and visceral fat-dependent impact of OSA on LFA. To test that hypothesis, we measured OSA, LFA, and VFA simultaneously and quantitatively.

## Methods

### Participants

We retrospectively surveyed 449 consecutive adults (≥20 years old) who were admitted to Kyoto University Hospital for polysomnography on suspicion of OSA between October 2008 and August 2010. Excluded were 97 individuals who met the following exclusion criteria: being administered medicines known to cause LFA; being serum HBs antigen and/or HCV antibody positive; having blood brain natriuretic peptide levels >100 pg/ml [18]; the presence of known or CT-diagnosed steatohepatitis, liver cirrhosis, or liver disease other than fatty liver disease; or the presence of another clinically serious disease. We clinically recommend an unenhanced abdominal CT examination for those suspected to have OSA to check the VFA [19–21]. Those who agreed to our recommendation underwent abdominal CT between the day when participants were referred to our clinic with suspicion of OSA and the day when OSA therapy was started. The study protocol was approved by the Kyoto University Graduate School and Faculty of Medicine Ethics Committee (IRB approval number E-1307) and written informed consent was obtained from all participants.

### CT Scanning and measurements

Unenhanced transverse CT was performed with an Aquilion 64 CT system (Toshiba Medical Systems Corporation, Tochigi, Japan) running on 135 kVp, 440 mA, and a 0.5-s scan time. The abdomen from the top of the liver to the lower region of the umbilicus was scanned. In this study, LFA was represented as the CT values for liver (CT_LFA_) in continuous variables because of the negative correlation between LFA and CT_LFA_. It has been reported that the CT_LFA_ is best for prediction of LFA among several CT parameters [22]. According to the measurement method used in this report, the CT_LFA_ was determined by averaging the CT values of 12 regions of interest placed on the CT liver images reconstructed at 7 mm intervals. Each region of interest was determined as a circular area of 100 ± 5 mm^2^, and was placed to avoid apparent vessels in each liver parenchyma of the 12 sections defined by the modified Couinaud segmentation system (S1 Fig). Furthermore, evaluating liver fat content is frequently performed by adjusting for CT values of the spleen. The CT values for spleen parenchyma were determined by averaging the CT values of 3 regions of interest placed in the cross sections dividing the spleen into 4 equal parts. The interclass correlation coefficient for inter-reader comparisons was 0.99 for CT_LFA_ in a randomly selected sample of 30 participants. To confirm the results, we also evaluated LFA by the liver/spleen ratio [23–25]. VFA and subcutaneous fat accumulation (SFA) were each measured as areas in a CT image of the umbilical level [26] using an image analysis program (AZE Virtual Place 99, AZE of America, Ltd., Irvine, CA, USA) [27]. Visceral obesity (VO) was defined by VFA ≥ 100 cm^2^ according to criterion for both Japanese males and females (VO_100_) proposed by the Japan Society for the Study of Obesity [28], which has been adopted by the Ministry of Health, Labor, and Welfare of Japan [29].

### Polysomnography

Polysomnography was performed in the standard manner from 22:00 until 6:00 the following morning according to recommendations in the American Academy of Sleep Medicine’s (AASM’s) manual (SomnoStar pro, Cardinal Health, Dublin, OH, USA) [30]. Surface electrodes were attached using standard techniques to obtain an electrooculogram, electromyogram of the chin, and 12-lead electroencephalograph. Ventilation was monitored by inductive plethysmography (Respitrace QDC, Viasys Healthcare, Palm Springs, CA, USA). Airflow was monitored by a nasal pressure transducer and supplemented by an oronasal thermal sensor. Arterial oxygen saturation (SpO_2_) was monitored continuously with a pulse oximeter, and 4%ODI [31], %T<90, and the lowest SpO_2_ during sleep were determined as indices of hypoxia (intermittent hypoxia, burden of hypoxia, and degree of desaturation). Apnea was defined as a cessation of airflow for at least 10 seconds and hypopnea was defined as an abnormal respiratory event lasting at least 10 seconds with a reduction > 50% in airflow by nasal pressure as compared to baseline or with an oxygen desaturation of > 3% or an arousal, which was proposed as an alternative scoring rule for hypopnea by AASM [30] and is usually used in clinical practice in Japan [32–34]. The AHI and 4%ODI were defined as the average number of apnea and hypopnea or 4% oxygen desaturation episodes per hour over the total sleep time, respectively. AASM recommended another hypopnea rule defined by an abnormal respiratory event lasting at least 10 seconds with a reduction >30% in airflow or with an oxygen desaturation of >4%. Therefore, we also scored AHI throughout sleep time, during REM, and in the supine position in all participants by using hypopnea as defined by this rule (AHI_AASM_).

### Data collection

Fasting venous blood samples were taken in the morning after overnight polysomnography. Insulin resistance was evaluated based on the homeostasis model assessment index (HOMA-IR) calculated from fasting plasma glucose and fasting serum insulin levels. Basic clinical parameters such as clinical history, medications, drinking habit, and smoking status were obtained from questionnaires. Daily alcohol intake was calculated from kinds and quantity of liquor and frequency of drinking and was used as a continuous variable. These questionnaires and anthropometric measurements were conducted on the day of polysomnography. Obesity was defined by BMI ≥25 kg/m^2^ according to criterion for Japanese proposed by the Japan Society for the Study of Obesity [28], which has been adopted by Ministry of Health, Labor, and Welfare of Japan [35].

### Statistical analysis

Results are expressed as mean ± standard deviation or number of participants. Following testing for normality and equality of variance, continuous variables were compared by unpaired *t* test, Welch’s tests, or Mann-Whitney U tests, and categorical variables for male and female participants were compared by Fisher's exact test. Prevalence of fatty liver among Japanese males and females was 40% and 22%, respectively [36], and the ratio of prevalence of OSA among males to that among females was about 2.3 [37]. When calculated from these data, the sample size to achieve 80% power at a 5% significance level in detecting the sex difference in fatty liver of OSA patients was 248 (173 males and 75 females). In addition, clinic-based studies reported that the prevalence of OSA (AHI ≥ 5 h^-1^) was 67% [4] and that 84% of those with OSA and 64% of those without OSA had fatty liver [10]. From these data, the sample size for detecting the influences of OSA on fatty liver was estimated to be 162.

The correlations between CT_LFA_, serum aspartate aminotransferase (AST) values, or serum alanine aminotransferase (ALT) values and other independent variables were analyzed by Pearson’s or Spearman's correlation coefficients and then stepwise multiple regression analyses were performed using variables yielding a P-value < 0.10 by univariate analysis. Independent variables included into these analyses were age, BMI, neck circumference, waist circumference, systolic blood pressure, diastolic blood pressure, daily alcohol intake, use of lipid-lowering agents, current smoking, AHI, 4%ODI, %T<90, lowest SpO_2_ during sleep, arousal index, REM sleep, AHI during REM, supine sleep time, AHI in supine position, Epworth Sleepiness Scale score, VFA, SFA, serum C-reactive protein (CRP), triglycerides, HDL-cholesterol and LDL-cholesterol levels, fasting plasma glucose levels, and the homeostasis model assessment of insulin resistance. When two independent variables had very strong collinearity (γ > 0.80), each was entered into the multivariate analysis separately and the best-fit model was adopted. P-values < 0.05 were considered statistically significant. All analyses were performed using JMP 9.0.2 (SAS Institute, Inc., Cary, NC, USA).

## Results

### Inclusion criteria and characteristics of study participants

**Fig 1:**
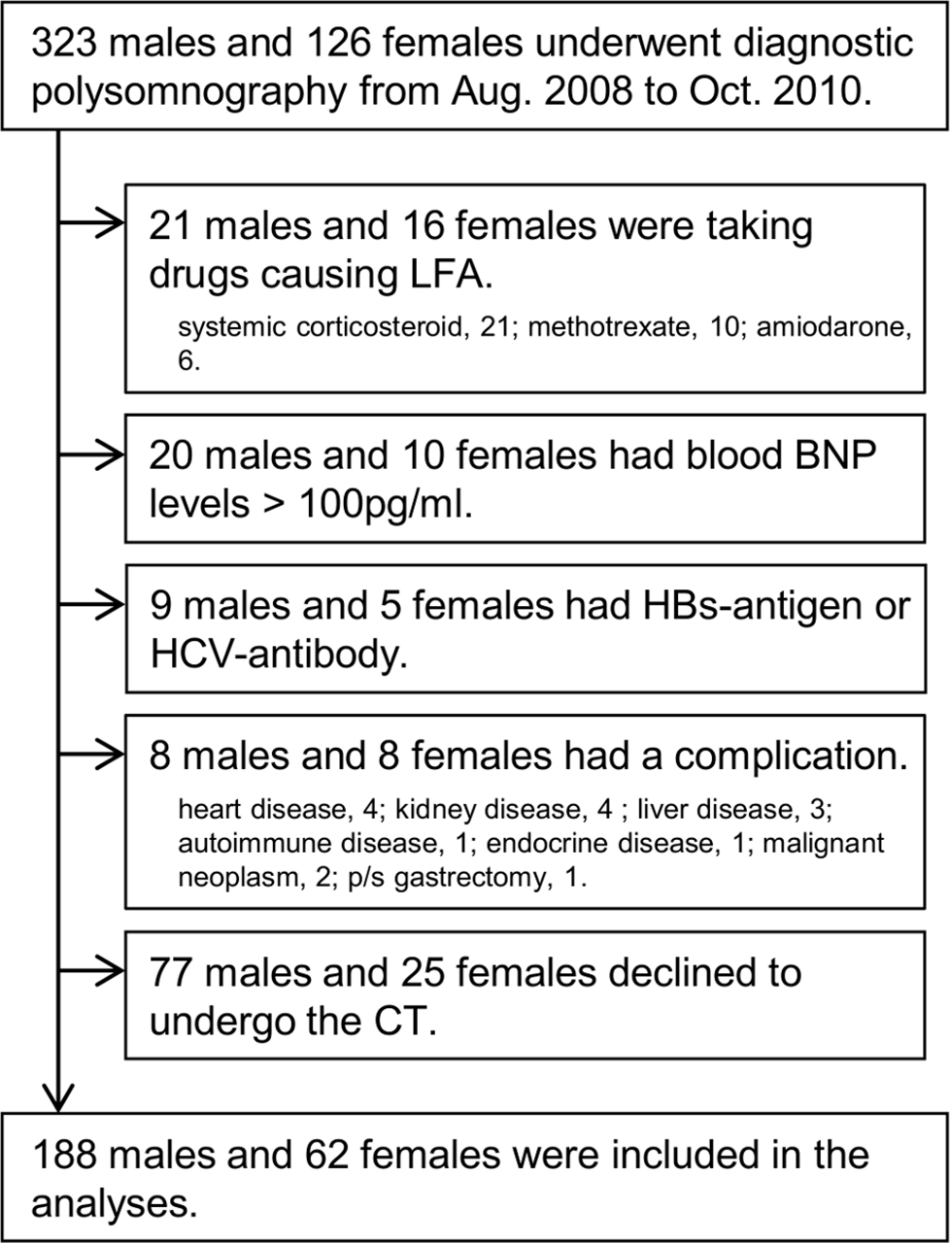
Flow chart of enrollment of study participants.

From the 449 adults who underwent diagnostic polysomnography during the study period, 97 participants were excluded due to the exclusion criteria and 102 declined the CT examination, leaving 250 to undergo abdominal CT (Fig 1). Between those who did and did not undergo CT examination, there were no significant differences in age, BMI, waist circumference, AHI, AST, and ALT. Characteristics of the 250 study participants (188 males, 62 females) are shown in Table 1. In this study population, age, BMI, and CT_LFA_ did not differ between males and females. Of the participants, 89 (34.8%) were taking lipid-lowering agents [38]. Of the 62 females, 14 were premenopausal, 40 were postmenopausal (1 was receiving hormone-replacement therapy), and the menopausal condition in 8 was unknown.

**Table 1:** Characteristics of study participants.

Characteristics	All participants	Males	Females	P-value^*^
Number (%)	250	188 (75.2)	62 (24.8)	…
Age, y	57.3 ± 13.1	56.5 ± 13.7	59.7 ± 11.1	0.095
BMI, kg/m^2^	26.6 ± 4.9	26.5 ± 4.6	27.0 ± 5.8	0.480
Neck circumference, cm	39.8 ± 4.6	40.7 ± 4.2	37.2 ± 5.4	<0.001
Waist circumference, cm	96.7 ± 10.2	96.1 ± 9.3	98.6 ± 12.4	0.149
Systolic blood pressure, mmHg	125.8 ± 13.7	125.7 ± 14.0	126.0 ± 12.8	0.870
Diastolic blood pressure, mmHg	77.8 ± 10.1	78.7 ± 10.3	75.0 ± 9.1	0.012
Alcohol intake, g/d	13.0 ± 23.0	16.8 ± 25.3	1.6 ± 4.5	<0.001
User of lipid-lowering agents, n(%)	89 (35.6)	63 (33.5)	26 (41.9)	0.284
Current smoker, n (%)	36 (14.5)	32 (17.1)	4 (6.5)	0.039
*CT parameters*				
Time from PSG to CT, m	-0.3 ± 1.2	-0.2 ± 1.0	-0.5 ± 1.6	0.173
CT_LFA_, HU	54.1 ± 12.8	53.8 ± 12.0	55.1 ± 15.0	0.523
VFA, cm^2^	113.6 ± 60.6	118.1 ± 61.7	100.2 ± 55.8	0.044
SFA, cm^2^	160.4 ± 95.7	145.6 ± 82.9	205.2 ± 116.5	<0.001
*Sleep parameters*				
AHI, h^-1^	31.6 ± 20.1	34.0 ± 20.1	24.4 ± 20.0	0.001
4%ODI, h^-1^	23.8 ± 20.5	26.2 ± 20.6	16.7 ± 18.7	0.002
%T<90, %	13.3 ± 20.1	14.9 ± 20.6	8.7 ± 17.8	0.001
Lowest SpO_2_ during sleep, %	79.4 ± 10.1	79.0 ± 10.1	80.8 ± 9.9	0.119
Arousal Index, h^-1^	31.0 ± 16.1	33.3 ± 16.0	23.9 ± 14.2	<0.001
REM sleep, %	14.9 ± 6.2	14.8 ± 6.2	15.1 ± 6.1	0.760
AHI during REM, h^-1^	35.3 ± 23.7	34.4 ± 23.6	37.9 ± 24.1	0.318
Supine sleep time, %	69.0 ± 26.2	69.0 ± 26.7	69.0 ± 24.4	0.987
AHI in supine position, h^-1^	39.8 ± 24.1	43.3 ± 24.5	29.3 ± 23.1	<0.001
Epworth sleepiness scale score	9.8 ± 5.3	10.0 ± 5.3	9.0 ± 5.1	0.180
*Blood parameters*				
AST, IU/L	24.5 ± 12.0	24.8 ± 11.9	23.6 ± 12.3	0.204
ALT, IU/L	28.3 ± 20.3	29.7 ± 20.8	24.3 ± 18.3	0.011
CRP, mg/dL	0.15 ± 0.24	0.14 ± 0.22	0.20 ± 0.28	0.284
Triglycerides, mg/dL	141.6 ± 89.6	150.1 ± 94.3	115.7 ± 67.7	0.002
HDL-cholesterol, mg/dL	48.8 ± 12.3	47.1 ± 11.6	54.3 ± 12.8	<0.001
LDL-cholesterol, mg/dL	112.4 ± 30.7	113.3 ± 29.9	109.8 ± 33.3	0.448
Fasting plasma glucose, mg/dL	104.5 ± 26.6	104.4 ± 24.0	104.6 ± 33.5	0.968
HOMA-IR	2.82 ± 3.17	2.71 ± 2.88	3.17 ± 3.92	0.772

All values are number (percentage) or mean ± standard deviation.

^*^Males vs. females.

A lower CT_LFA_ means higher liver fat accumulation. Among users of lipid-lowering agents [38], 7 were taking pioglitazone, 76 statins, 4 eicosapentaenoic acid, 5 ezetimibe, 6 tocopherol, and 11 telmisartan; among them, 20 were taking 2 of these medicines.

*Abbreviations*: BMI, body mass index; CT, computed tomography; PSG, polysomnography; CT_LFA_, CT values for liver; VFA, visceral fat accumulation; SFA, subcutaneous fat accumulation; AHI, apnea-hypopnea index; ODI, oxygen desaturation index; %T<90, percentage of time spent with SpO_2_ below 90% to total sleep time; SpO_2_, oxygen saturation measured by pulse oximetry; REM, rapid eye movement; AST, aspartate aminotransferase; ALT, alanine aminotransferase; CRP, C-reactive protein; HOMA-IR, homeostasis model assessment of insulin resistance.

### Association between OSA and CT_LFA_ by univariate regression analyses in male and female participants

AHI was negatively correlated with CT_LFA_ in all participants (Table 2). Although the number of females might not be sufficient for separate analyses, in a sex-specific analysis AHI was negatively correlated with CT_LFA_ in males (γ = −0.288, P < 0.001) but not in females (γ = −0.011, P = 0.933). In females, even when limited to the postmenopausal females without hormone-replacement therapy (n = 39), AHI was not correlated with CT_LFA_.

**Table 2:** Correlation coefficients of CT_LFA_ in all participants and separately in males, and females.

	All participants	Males	Females
Age, y	0.26^‡^	0.25^‡^	0.27^†^
BMI, kg/m^2^	-0.61^‡^	-0.63^‡^	-0.58^‡^
Neck circumference, cm	-0.41^‡^	-0.35^‡^	-0.55^‡^
Waist circumference, cm	-0.55^‡^	-0.54^‡^	-0.56^‡^
Systolic blood pressure, mm Hg	-0.17^†^	-0.17^†^	-0.20
Diastolic blood pressure, mm Hg	-0.22^‡^	-0.21^†^	-0.27^†^
Alcohol intake, g/d	-0.02	-0.02	0.16
^§^Use of lipid-lowering agents	-0.05	-0.13^*^	0.12
^§^Current smoking	-0.12^*^	-0.12	-0.08
VFA, cm^2^	-0.49^‡^	-0.49^‡^	-0.53^‡^
SFA, cm^2^	-0.49^‡^	-0.59^‡^	-0.43^‡^
AHI, h^-1^	-0.21^‡^	-0.29^‡^	-0.01
4%ODI, h^-1^	-0.24^‡^	-0.29^‡^	-0.09
%T90, %	-0.26^‡^	-0.31^‡^	-0.11
Lowest SpO_2_ during sleep, %	0.13^†^	0.20^†^	-0.05
Arousal Index, h^-1^	-0.03	-0.04	0.03
REM sleep, %	-0.03	0.04	-0.18
AHI during REM, h^-1^	-0.22	-0.33^‡^	0.04
Supine sleep time, %	0.12^*^	0.06	0.28^†^
AHI in supine position, h^-1^	-0.21^‡^	-0.27^‡^	-0.05
Epworth sleepiness scale score	-0.17^†^	-0.16^†^	-0.16
CRP, mg/dL	-0.30^‡^	-0.28^‡^	-0.34^†^
Triglycerides, mg/dL	-0.41^‡^	-0.42^‡^	-0.44^‡^
HDL-cholesterol, mg/dL	0.36^‡^	0.38^‡^	0.33^†^
LDL-cholesterol, mg/dL	-0.13^†^	-0.16^†^	-0.06
Fasting plasma glucose, mg/dL	-0.22^‡^	-0.22^†^	-0.23^*^
HOMA-IR	-0.40^‡^	-0.44^‡^	-0.35^†^

^*^P <0.10

^†^P <0.05

^‡^P <0.001, which indicate variables entered into the multivariate regression analyses.

^§^Correlation coefficients of these variables are indicated by Spearman's ρ, and the others are indicated by Pearson's γ. CT_LFA_ is negatively correlated with liver fat accumulation.

*Abbreviations*: CT_LFA_, CT values for liver; BMI, body mass index; VFA, visceral fat accumulation; SFA, subcutaneous fat accumulation; AHI, apnea-hypopnea index; ODI, oxygen desaturation index; %T<90, percentage of time spent with SpO_2_ below 90% to total sleep time; SpO_2_, oxygen saturation measured by pulse oximetry; REM, rapid eye movement; CRP, C-reactive protein; HOMA-IR, homeostasis model assessment of insulin resistance.

As the Japanese criterion for visceral obesity is VFA greater than 100 cm^2^ (VO_100_), we firstly conducted regression analyses of the participants stratified according to the presence or absence of VO_100_. In the males, AHI was not significantly correlated with CT_LFA_ in males with VO_100_ (n = 113) (γ = −0.126, P = 0.182) but was significantly correlated with CT_LFA_ in males without VO_100_ (n = 75) (γ = −0.376, P < 0.001). In the females, none of the OSA-related indices was correlated with CT_LFA_ whether VFA was over or under 100 cm^2^ (S1 Table).

### Multivariate regression analyses for CT_LFA_ in males with and without VO_100_

**Fig 2:**
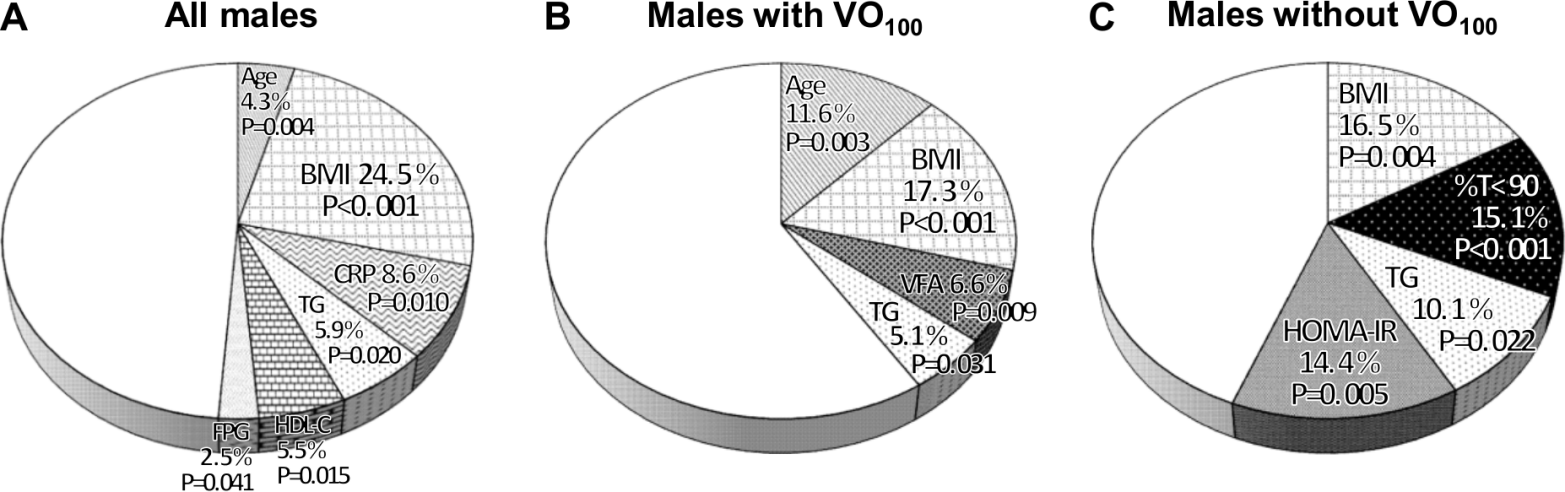
Coefficients of determination (R^2^) in stepwise multiple regression models for CT_LFA_ in all males and males stratified according to VO_100_. (A) All males, (B) males with VO_100_, and (C) males without VO_100_. Variables entered into the stepwise regression analyses were selected from age, BMI, neck circumference, waist circumference, systolic and diastolic blood pressures, alcohol intake, use of lipid-lowering agents, current smoking, VFA, SFA, AHI, 4%ODI, %T<90, lowest SpO_2_, arousal index, REM sleep, AHI during REM, supine sleep time, AHI in supine position, Epworth sleepiness scale score, CRP, triglycerides (TG), HDL-cholesterol (HDL-C), LDL-cholesterol, fasting plasma glucose (FPG), and HOMA-IR when yielding a P-value <0.10 by univariate analysis.

When multivariate analyses were conducted in male participants, none of the OSA-related factors was independently associated with CT_LFA_ in the total male participants (Table 3, Fig 2A), male OSA participants (AHI ≥ 10 h^-1^, n = 164), or in male control participants (AHI < 10 h^-1^, n = 24). In similar analyses, %T<90 was an independent determinant of CT_LFA_ not in males with VO_100_ but in males without VO_100_ (R^2^ = 15.1%, P < 0.001) (Table 4, Fig 2B and 2C). When 4%ODI was separately entered into the stepwise analysis for males without VO_100_ because of its strong collinearity (γ = 0.830 with %T<90), it remained in the model as an independent determinant of CT_LFA_ (R^2^ = 8.8%, P = 0.013; Table 4 model B). Moreover, we used AHI_AASM_ instead of the AHI that we had adopted. In this case, AHI_AASM_ was entered into the analysis separately from 4%ODI and comprised the stepwise model for CT_LFA_ in males without VO_100_ in almost the same pattern as produced by 4%ODI (R^2^ of AHI_AASM_ = 7.6%, cumulative R^2^ of the model = 50.9%). When the liver/spleen ratio was adopted as a parameter representing LFA, both of these indices of OSA-related hypoxemia were independent determinants of LFA (%T<90, R^2^ = 10.5%, P = 0.003; or 4%ODI, R^2^ = 6.3%, P = 0.019). Since there was a significant correlation not between alcohol intake and CT_LFA_ but between the use of lipid-lowering agents and CT_LFA_, we excluded users of lipid-lowering agents (n = 20) or non-OSA participants (AHI < 10 h^-1^, n = 11) from the males without VO_100_ and performed a similar analysis. As a result, the independent contributions of %T<90 to CT_LFA_ did not change even in these subanalyses.

**Table 3:** Stepwise multiple regression models for CT_LFA._

	All participants (n = 250)			Males (n = 188)			Females (n = 62)		
*Variables*	β	R^2^, %	*P* value	β	R^2^, %	*P* value	β	R^2^, %	*P* value
Age	0.11	2.7	0.031	0.17	4.3	0.004	−	−	−
BMI	-0.44	27.0	<0.001	-0.39	24.5	<0.001	-0.39	22.3	<0.001
Neck circumference	−	−	−	−	−	−	-0.32	17.6	0.002
CRP	-0.11	3.3	0.023	-0.18	8.6	0.010	−	−	−
Triglycerides	-0.21	8.7	<0.001	-0.14	5.9	0.020	-0.33	14.5	<0.001
HDL-cholesterol	0.12	4.2	0.026	0.14	5.5	0.015	−	−	−
Fasting plasma glucose	-0.11	2.4	0.022	-0.11	2.5	0.041	−	−	−
*Cumulative R* ^*2*^	…	48.5	…	…	51.2	…	…	54.4	…

Variables entered into the stepwise regression analyses were indicated in Table 2 by variables yielding P-value <0.10; only variables left in one or more of the models are shown in this table. Minus sign means the variable was not selected through univariate or stepwise selection procedures. CT_LFA_ is negatively correlated with liver fat accumulation.

*Abbreviations*: CT_LFA_, CT values for liver; β = standard regression coefficient; R^2^ = coefficient of determination; BMI, body mass index; CRP, C-reactive protein.

**Table 4:** Stepwise multiple regression models for CT_LFA_ in males with and without VO_100._

	Males with VO_100_			Males without VO_100_ (n = 75)					
	(n = 113)			Model A			Model B		
*Variables*	β	R^2^, %	*P* value	β	R^2^, %	*P* value	β	R^2^, %	*P* value
Age	0.26	11.6	0.003	−	−	−	−	−	−
BMI	-0.33	17.3	<0.001	-0.29	16.5	0.004	-0.31	17.9	0.003
%T<90	−	−	−	-0.31	15.1	<0.001	…	…	…
4%ODI	−	−	−	…	…	…	-0.22	8.8	0.013
VFA	-0.21	6.4	0.009	−	−	−	−	−	−
Triglycerides	-0.17	5.1	0.031	-0.21	10.1	0.022	-0.23	11.0	0.018
HOMA-IR	−	−	−	-0.26	14.4	0.005	-0.26	13.9	0.011
*Cumulative R* ^*2*^	…	40.4	…	…	56.0	…	…	51.5	…

Variables entered into the stepwise regression analyses were selected from age, BMI, neck circumference, waist circumference, systolic and diastolic blood pressures, alcohol intake, use of lipid-lowering agents, current smoking, VFA, SFA, AHI, 4%ODI, %T<90, lowest SpO_2_, arousal index, REM sleep, AHI during REM, supine sleep time, AHI in supine position, Epworth sleepiness scale score, CRP, triglycerides, HDL-cholesterol, LDL-cholesterol, fasting plasma glucose, and HOMA-IR when yielding a P-value <0.10 by univariate analysis; %T<90 (Model A) and 4%ODI (Model B) were entered into the analyses separately for their strong collinearity; only variables left in one or more of the models are shown in this table. Minus sign means the variable was not selected through univariate or stepwise selection procedures. CT_LFA_ is negatively correlated with liver fat accumulation.

*Abbreviations*: VO_100_, visceral obesity (VFA ≥100 cm^2^); β = standard regression coefficient; R^2^ = coefficient of determination; BMI, body mass index; %T<90, percentage of time spent with SpO_2_ below 90% to total sleep time; ODI, oxygen desaturation index; VFA, visceral fat accumulation; HOMA-IR, homeostasis model assessment index of insulin resistance.

### VFA threshold level of the association between OSA and CT_LFA_

To determine the threshold of VFA levels at which %T<90% was a significant determinant of CT_LFA_ in males, stepwise models were examined for VFAs of 115 cm^2^, 130 cm^2^, and 145 cm^2^. From these analyses, %T<90 was a significant determinant of CT_LFA_ until VFA reached 130 cm^2^ (VO_130_).

### Interaction of obesity with the association between OSA and CT_LFA_

When participants were stratified by the presence or absence of obesity defined by Japanese criterion (BMI ≥ 25 kg/m^2^) [28] instead of VO, independent determinants of CT_LFA_ were age, BMI, VFA, and triglycerides in males with obesity (n = 110); BMI, HDL-cholesterol, and HOMA-IR in males without obesity (n = 78); BMI, triglycerides and HOMA-IR in females with obesity (n = 35); and neck circumference in females without obesity (n = 27). However, none of the OSA-related indices was detected as a determinant of CT_LFA_ in these stratified groups (S2 and S3 Tables).

### Association between OSA and serum transaminase values in univariate and multivariate regression analyses

**Fig 3:**
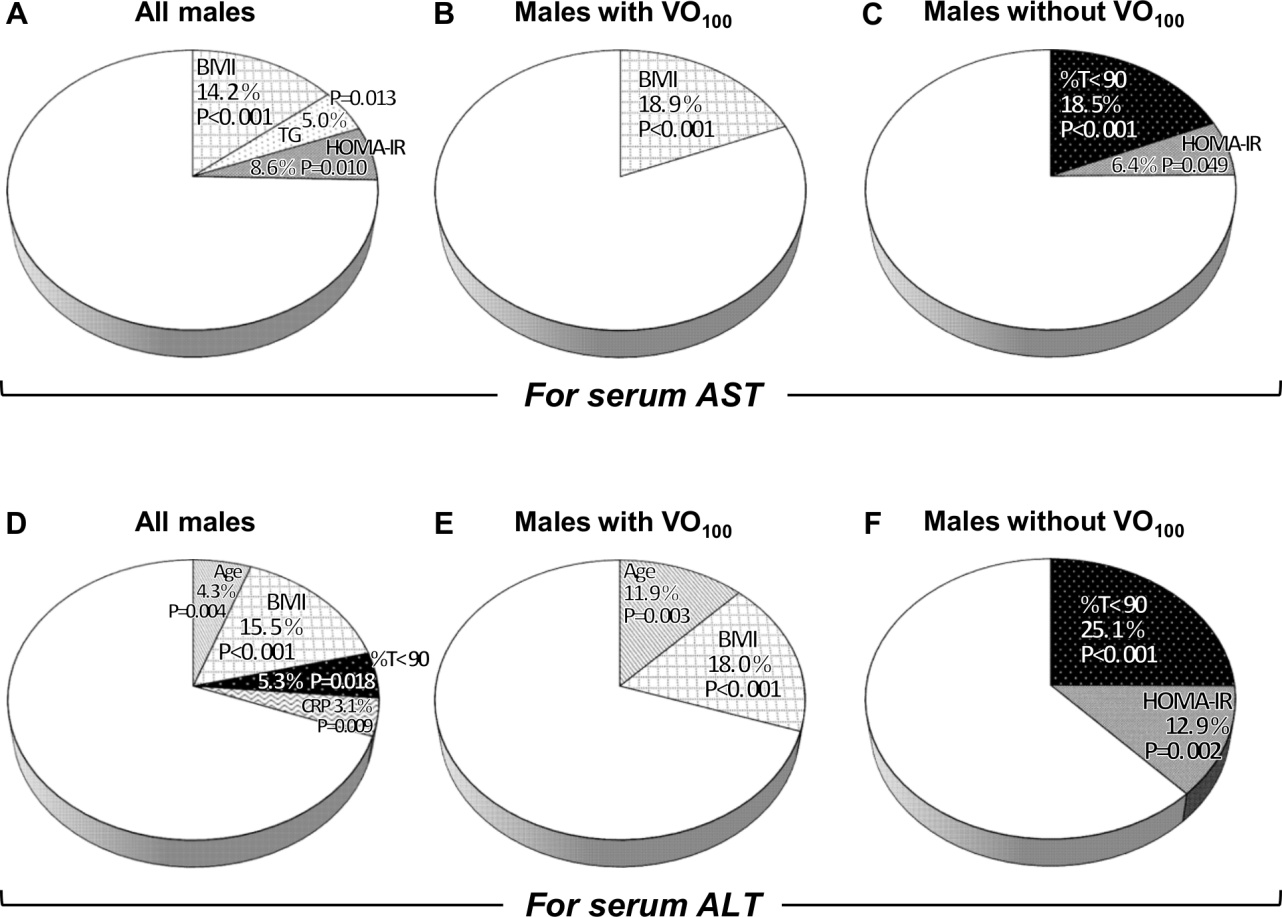
Coefficients of determination (R^2^) in stepwise multiple regression models for serum transaminase values in all males and males stratified according to VO_100_. Serum AST and ALT values in all males (A, D), in males with VO_100_ (B, E), and in males without VO_100_ (C, F). Variables entered into the stepwise regression analyses were selected from age, BMI, neck circumference, waist circumference, systolic and diastolic blood pressures, alcohol intake, use of lipid-lowering agents, current smoking, VFA, SFA, AHI, 4%ODI, %T<90, lowest SpO_2_, arousal index, REM sleep, AHI during REM, supine sleep time, AHI in supine position, Epworth sleepiness scale score, CRP, triglycerides (TG), HDL-cholesterol (HDL-C), LDL-cholesterol, fasting plasma glucose (FPG), and HOMA-IR when yielding a P-value <0.10 by univariate analysis.

In the males, while AHI was significantly correlated with serum AST and ALT values, multiple regression analyses showed that %T<90 was an independent determinant of not AST but ALT values (Fig 3A and 3D). However, in males without VO_100_, %T<90 was an independent determinant of AST and ALT values (Fig 3C and 3F). This independent contribution of %T<90 to transaminase values did not change even after non-OSA participants, excessive drinkers, or users of lipid-lowering agents were excluded from the males without VO_100_.

In the females, OSA was not correlated with serum AST or ALT values in the total female group, in postmenopausal females without hormone-replacement therapy, in females with VO_100_, or in females without VO_100_.

## Discussion

In this study, we demonstrated significant associations among OSA, LFA, sex, and VFA. Such associations have not been fully investigated in previous studies. That is, the %T<90 (rather than 4%ODI), namely sleep-associated hypoxia, was a major independent risk factor for LFA evaluated by CT_LFA_ but only in males with VFA < 130 cm^2^. Moreover, %T<90 was associated with serum ALT values regardless of VO criteria. However, such associations between OSA and LFA or serum transaminase values were not observed in females at all.

### Impact of OSA on LFA and sex difference

There are two main putative mechanisms by which OSA increases LFA, one of which is the activation of triglyceride biosynthesis in liver through a pathway from hypoxia-inducible factor-1α (HIF-1α) [13, 14, 39], and the other is the increase in serum free fatty acid values observed in OSA patients [40, 41]. Ryan et al. reported that HIF-1α activity was induced by exposure to sustained hypoxia in a dose-dependent manner but not by exposure to intermittent hypoxemia [42]. In our study of humans, 4%ODI representing intermittent hypoxemia was also associated with LFA, but its effect on the determinants was smaller than %T<90 representing sleep-associated hypoxia (Table 4, Models A and B). Thus, sleep-associated hypoxia rather than intermittent hypoxemia seems to have the greater influence on LFA, as was also reported recently [12]. In a mouse model exposed to intermittent hypoxemia (12 times/h or 60 times/h), intermittent-hypoxemia exposure caused not only intermittent-hypoxemic effects but also sustained-hypoxic effects on the liver and fat tissues, and the higher frequencies of intermittent hypoxemia produced more severe effects [43].

One of the important findings of this study was a sex difference in the association between OSA and LFA. Few studies have investigated sex differences in the association between OSA and LFA. In this study, although there was no significant difference in age, BMI, and CT_LFA_ between males and females, an independent association between OSA and LFA was observed only in males but not in females. One reason for this may be the influence of estrogen, which was reported to inhibit HIF-1α and key enzymes for triglyceride biosynthesis [44, 45]. However, even when excluding premenopausal females in this study, the result did not change. In addition to the low prevalence of severe OSA in females (Table 1), the age of onset of OSA is later in females than in males, with the prevalence of OSA increasing in females after menopause. Thus, females may have less cumulative exposure to OSA than men with similar severities of OSA [46]. In addition, sex differences in unmeasured health behaviors such as diet or exercise as well as changes over time in risk factors such as alcohol intake and obesity cannot be excluded as causes of the sex difference in OSA-associated LFA. Since the number of females was small in this study, the cumulative effects of OSA and the effects of estrogen on OSA-related diseases including LFA should be studied further in large-scale studies.

### Visceral fat-dependent impact of OSA on LFA

The second important finding of this study is that an association between OSA and LFA was observed only when VFA was less than 130 cm^2^. Similarly, the improvement by continuous positive airway pressure therapy in insulin sensitivity of OSA patients was reported to be much greater in non-obese than in obese patients [47]. In addition, it was the VFA rather than the BMI that impacted the association between OSA and LFA in our study. This may be explained by the dose-dependent impact of VFA, which is the main source of free fatty acids and is more greatly associated with LFA than SFA or BMI [1, 15, 48]. Tatsumi et al. reported that OSA was not a risk factor for fatty liver in Japanese, who had an average VFA of 156 cm^2^ [4]. These results, including ours, suggest that a large VFA, which would be greater than 130 cm^2^ in Asians, overwhelms OSA in terms of its impact on LFA. Similar findings were also observed in animal examinations. LFA at baseline was much greater in obese mice than in lean mice, and it took a much longer time to identify a significant increase in LFA by intermittent hypoxia in the existing fatty liver of obese mice [13, 14]. Recently, Minville et al. reported that %T<90, HOMA-IR, and serum triglyceride values were independently associated with fatty liver, similar to our results [12]. However, contrary to our findings, they found an association between OSA and LFA only in morbidly obese patients. Although the SteatoTest, which they used to determine fatty liver, is convenient and well-validated, it is a scoring system consisting of several blood markers such as triglycerides and glucose and, therefore, potentially strongly correlates with HOMA-IR and triglycerides and tends to be influenced by obesity [49]. CT evaluation of fatty liver is more sensitive, specific, and quantitative [22, 23]; therefore, we believe our findings would also be accurate and that CT would be more suitable for evaluation of fatty liver than biomarkers.

### Methods in CT measurements

In this study, the CT_LFA_ was measured in all participants by a single well-calibrated method that allowed the evaluation of the whole burden of LFA by setting regions of interest in each anatomical section of the liver [22]. As mentioned in Methods, CT_LFA_ was reported to be best for prediction of LFA among several CT parameters. In addition, we confirmed the results by the liver/spleen ratio, a method that is also frequently used to measure LFA [23–25]. Results using the two different methods were almost the same. Therefore, it can be considered that the results of this study are accurate.

### Study limitations

Limitations of this study should be mentioned. Firstly, we cannot know causality or the mechanisms of our findings because this was a cross-sectional observational study. This study was done at the time of diagnosis of OSA, so the duration of OSA was unclear. Secondly, we measured LFA using CT images instead of biopsy specimens. CT measurement can be used for evaluation of LFA, particularly in those who should avoid an invasive biopsy [1], and also can evaluate the whole burden of LFA regardless of heterogeneously spreading fat. Thirdly, our study also included fewer females than the calculated sample size and OSA was less severe in the females than in the males. These factors might have caused β errors in analyses concerning female participants. Therefore, the results could have been different with a greater number of postmenopausal female participants. However, significant differences in the associations among VFA, LFA, and OSA between males and females in this study may support previous knowledge that the prevalence of LFA and the pattern of body fat distribution differ by sex [1, 28]. Fourthly, although we adopted obesity criteria for Japanese proposed by Japan Society for the Study of Obesity (VFA ≥100 cm^2^ or BMI ≥25 kg/m^2^, irrespective of sex) [34], some studies implied that the cutoff value of VFA for Japanese females should be less than 100 cm^2^ [50, 51]. Larger-scale and more recent studies supported the Japan Society's criteria [52, 53], but there would still be room to examine the cutoff values for VFA in fatty liver especially for Japanese females. Fifthly, self-reported sleep duration and quality, which were recently reported to be associated with fatty liver independently of OSA [54], were not considered. In future investigations of fatty liver, sleep duration and quality should be evaluated objectively in a home setting. Finally, ethnic differences as shown between BMI and the prevalence of OSA should be further studied.

## Conclusions

OSA-related hypoxemia (%T<90) was a significant risk factor for LFA in patients with a VFA of 130 cm^2^ or less and for an elevation of ALT regardless of VO criteria. However, these associations were observed only in males, which might be the same phenomenon as the sex difference observed in the cardiovascular prognosis of OSA patients. These results suggested that treatment for male OSA with hypoxemia during sleep might be warranted from the viewpoint of preventing LFA in males without overt VFA and liver dysfunction.

## Supporting Information

S1 Fig Modification of Couinaud segmentation system. Each ROI for measurement of attenuation was placed in the liver parenchyma of each section. (A) Level of right hepatic vein; (B) level of umbilical portion of left portal vein; (C) level of posterior branch of right portal vein. Abbreviations: HV, hepatic vein; IVC, inferior vena cava, UP, umbilical portion; Post.PV, posterior branch of right portal vein.
(TIF)

S1 File Study protocol. (DOC)

S2 File Study protocol in the original language. (DOC)

S1 Table Stepwise multiple regression models for CT_LFA_ in females with and without VO_100_. (DOC)

S2 Table Stepwise multiple regression models for CT_LFA_ in males with and without obesity (BMI ≥ 25 kg/m^2^). (DOC)

S3 Table Stepwise multiple regression models for CT_LFA_ in females with and without obesity (BMI ≥ 25 kg/m^2^). (DOC)
