# Supplementary figures and images for: Impact of Obstructive Sleep Apnea on Liver Fat Accumulation According to Sex and Visceral Obesity
Source: PLoS One. 2015 Jun 15;10(6):e0129513. doi: 10.1371/journal.pone.0129513 (PMC4468199; doi:10.1371/journal.pone.0129513)

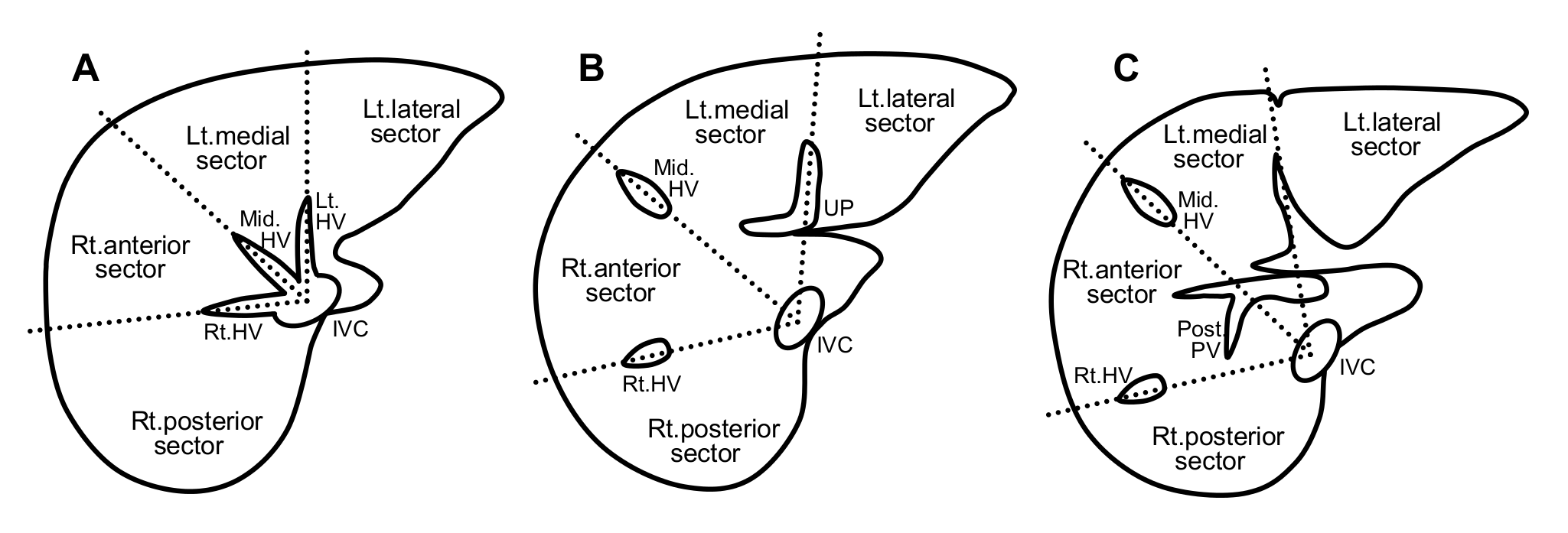

Supplement: S1 Fig — Each ROI for measurement of attenuation was placed in the liver parenchyma of each section. (A) Level of right hepatic vein; (B) level of umbilical portion of left portal vein; (C) level of posterior branch of right portal vein. Abbreviations: HV, hepatic vein; IVC, inferior vena cava, UP, umbilical portion; Post.PV, posterior branch of right portal vein. (TIF) [file pone.0129513.s001.tif]
