# Supplementary material for: Impact of Obstructive Sleep Apnea on Liver Fat Accumulation According to Sex and Visceral Obesity
Source: PLoS One. 2015 Jun 15;10(6):e0129513. doi: 10.1371/journal.pone.0129513 (PMC4468199; doi:10.1371/journal.pone.0129513)
Supplement: S1 File — (DOC) [file pone.0129513.s002.doc]

**Study protocol**

**STUDY TITLE:** Investigation of fatty liver in sleep apnea patients with metabolic syndrome or visceral obesity and effects of CPAP treatment on their fatty liver.

**BACKGROUND:** It is said that one of three or four adults have fatty liver today [1-4]. It has become clear that simple steatosis, which is regarded as a benign condition, can progress to steatohepatitis, liver cirrhosis or hepatocellular carcinoma [5,6]. On the other hand, visceral fat accumulation and insulin resistance are related to the progression of fatty liver diseases [7-9]. Diagnosis of the fatty liver disease requires histopathology by liver biopsy. However, it is difficult to perform a liver biopsy in all patients with fatty liver because of its invasiveness and high prevalence of the disease. Therefore, easier and less invasive methods are desirable to diagnose and evaluate the disease. Among them, computed tomography (CT) has been shown to be useful to evaluate fatty liver [10-13].

Obstructive sleep apnea (OSA) causes repetitive intermittent hypoxia, activation of sympathetic nervous system and oxidative stress, and OSA is regarded as a risk factor for various complications such as cardiovascular diseases, hypertension and insulin resistance, etc. Associations among OSA, visceral fat accumulation and insulin resistance is a current topic [14-16], it has been indicated that nocturnal hypoxemia is a risk factor of liver enzyme abnormality [17,18] or steatohepatitis [19], and it has been shown in animal examinations that the liver fat content increases after exposure to intermittent hypoxia [20]. In addition, it was reported that visceral fat accumulation, insulin resistance and increased values of liver enzymes of OSA patients improved through continuous positive airway pressure (CPAP) treatment [14,21,22], and it is suggested by a study containing a small sample size that fatty liver may improve by CPAP treatment [23]. On the other hand, some studies seemed to deny the association among OSA, visceral fat accumulation and fatty liver [17,24].

Thus, it is still unclear how OSA and visceral fat accumulation influence fatty liver and how CPAP treatment for OSA influences fatty liver. Images acquired from CT performed at our hospital to evaluate metabolic syndrome or visceral fat accumulation usually include liver images. We have decided to evaluate fatty liver as well as visceral fat accumulation by using these CT images.

**OBJECTIVE:** To investigate associations between indices of OSA severity and the CT values for liver while taking into consideration the influences on it of visceral fat accumulation and other parameters in the participants who underwent polysomnography (PSG) on suspicion of OSA and underwent plain abdominal CT as a regular examination to evaluate metabolic syndrome or visceral fat accumulation at our hospital. In addition, in patients who started CPAP for OSA diagnosed by baseline PSG, continued the therapy at least for 6 months, and had metabolic syndrome, visceral obesity or fatty liver at the baseline, we will perform plain abdominal CT to evaluate the condition of current disease and the impact of the therapy. In addition, we will investigate the change of the CT values for liver, visceral fat accumulation and other parameters before and after CPAP treatment.

***1. Study Design***

Cross-sectional study, Cohort Study

***2. Study Period***

**1) Registration Period**

For 2 years from the day when Kyoto University Graduate School and Faculty of Medicine Ethics Committee admits the protocol.

**2) Follow-up Period**

In the follow-up study, study subjects are those who started CPAP for OSA and are visiting our hospital at the follow-up examination.

***3. Objects Selection***

**1) Place of Selection**

Kyoto University Hospital

**2) Selection Criteria**

*Inclusion Criteria*

In the cross-sectional study, adult subjects (≥ 20 y) who were newly suspected to have OSA by symptoms such as snoring or dyspnea during sleep and underwent PSG as a diagnostic examination at our hospital and underwent abdominal CT to decide whether he (she) had visceral obesity or metabolic syndrome shall be included. In the longitudinal study to investigate the influence of CPAP therapy, of the subjects included in the cross-sectional study, those who started CPAP for OSA, continue the therapy at least for 6 months, are visiting our hospital, had metabolic syndrome, visceral obesity or fatty liver at the baseline and consent to the abdominal CT examination shall be included.

*Exclusion Criteria*

- Subjects treated for sleep disordered breathing at the time of diagnostic PSG.
- Subjects with liver disease, infection of hepatitis virus, a congestive condition or another clinically serious disease.
- Subjects taking drugs causing fatty liver such as methotrexate or amiodarone.
- Subjects who had difficulty in deciding whether he (she) agreed with the enrollment in this study for himself (herself).

**3) Target sample size**

- Those who underwent diagnostic PSG at our hospital and met the inclusion criteria during the study period.

***4. Baseline and Follow-up examination***

Severity of OSA shall be evaluated by the baseline PSG. The CT values for liver shall be measured in the images of liver contained in abdominal CT performed to evaluate the amount of visceral fat. In the follow-up examination, abdominal CT shall be performed in those who started CPAP according to the diagnosis of OSA by the baseline PSG, continue the therapy at least for 6 months, are visiting our hospital, had metabolic syndrome, visceral obesity or fatty liver at the baseline examination and consent to the abdominal CT examination. Anthropometry, biochemical tests and measurement of humoral factors shall be also performed at the baseline and follow-up examination. Treatment states for OSA by CPAP shall be evaluated by the data recorded in memory cards inserted into the CPAP devices.

***5. Items of examination***

Measurement place: Kyoto University Hospital

**1) Baseline examinations at the baseline PSG**

Characteristics

- Age, Sex, Height, Weight, Body mass index, Waist circumference, Neck circumference, Blood pressure
- Past history, Smoking history, Alcohol intake, Other treatment or medication

QOL, Questionnaire on sleep status

- Epworth Sleepiness Scale (ESS): Evaluation of subjective sleepiness
- Medical Outcomes Study 36-Item Short Form Health Survey (SF-36): Evaluation of general health status
- Pittsburgh Sleep Quality Index (PSQI): Evaluation of sleep quality
- Morningness-Eveningness questionnaire: Evaluation of sleep phase
- Calgary Sleep Apnea Quality of Life Index (SAQLI): Evaluation of disease-specific health status
- British Medical Research Council Dyspnea Scale (MRC): Evaluation of subjective dyspnea
- Hospital Anxiety and Depression Scale (HADS): Evaluation of anxiety and depression
- Physical Activity: Evaluation of daily activity

PSG

Plain abdominal CT

Plain abdominal CT, which are usually performed at our hospital within the health insurance, shall be performed in males ≥ 20 y or females ≥ 40 y who have possibility of having metabolic syndrome or visceral obesity and agree to undergo abdominal CT.

Fasting blood test, Morning urine test

Blood cell count, Liver function, Renal function, Glucose metabolism, Lipid metabolism, Inflammation markers, Hormones and humoral factors, etc.

Artery blood gas

Electrocardiogram, Spirometry

* All of these but are the routine examinations performed in any subjects at the time of admission for PSG for evaluation OSA.

***2) After introduction of CPAP (6 months or more)***

Characteristics (Age, Height, Weight, Body mass index, Waist circumference, Neck circumference, Blood pressure, treatment or medication)

Data recorded in the CPAP memory cards (Apnea hypopnea index (AHI), Usage adherence, etc.)

Plain abdominal CT (those who had metabolic syndrome, visceral obesity or fatty liver at the baseline and agree to undergo the CT examination)

Fasting blood test (Liver function test, lipid profile, etc.)

***6. Possible adverse events by the intervention and measurements***

In the examinations, possible invasions are injury by blood sampling and radiation exposure by plain abdominal CT. However, plain CT for evaluation of visceral fat is usually performed at our hospital under the agreement, so there is no additional radiation exposure for this study. We necessarily advise those with abnormality in the first CT examination to reduce their weight in addition to CPAP treatment, and follow-up CT shall be performed within usual medical practice to evaluate effect of these treatments and decide further treatment strategy after they agree to undergo the examination. Therefore, there is no unnecessary radiation exposure. CPAP treatment as an intervention is applied for OSA patients meeting criteria of CPAP introduction within usual medical practice regardless of the study, so there is no additional invasion or adverse event according to the study.

***7. Outlines of the study analysis as an epidemiological survey***

- To investigate by using multivariate analysis whether indices of OSA severity (AHI, Percent sleep time with oxygen saturation (SpO_2_) under 90%; Mean SpO_2_ during sleep, Lowest SpO_2_ during sleep, Arousal index, etc.) correlate with the CT values for liver independently from the other related factors.
- To investigate whether improvement of indices of OSA severity by CPAP treatment correlate with change of the CT values for liver or change of visceral fat area during CPAP therapy independently from the other related factors.

***8. Protection of personal information***

To protect participants' privacy, any personal data are anonymized and it is made impossible to connect data to individuals. An anonymous database and a correspondence list to relate registration numbers specific for this study to participants' identification numbers shall be stored separately in the computer locked by the password known to only the researcher in the laboratory, and only the principal investigator and coworkers can access the data. These data shall be deleted properly after the analyses.

***9. Explanation and agreement of the study to participants, and ethical consideration for them***

This research will be conducted according to “the Declaration of Helsinki,” “the Ethical Guidelines for Epidemiological Research,” and “the Ethical Guidelines for Clinical Research.” The researcher or the medical attendant will explain to the participants about the study and receive the written informed consent from him or her. One of the two same documents about the agreement shall be stored by the researcher. Participants shall be explained that they can cancel their participation anytime and that they do not receive any disadvantage when they do not participate in this study.

***10. Burden of costs and honorarium***

1) The expense of examinations and CPAP treatment, which are necessary for evaluation of the disease condition and treatment for the disease and are under the national health insurance system, shall be done under the health insurance systems.

2) No honorarium to the participants shall be prepared.

***11. Lists of Researchers***

**Chief Researcher**

*Prof. Kazuo Chin*

The Department of Respiratory Care and Sleep Control Medicine, Graduate School of Medicine, Kyoto University, Kyoto, Japan

Tel. +8175-751-3852

**Co-Researchers**

*Dr. Toru Oga*

*Dr. Takefumi Hitomi*

*Dr. Chikara Yoshimura*

The Department of Respiratory Care and Sleep Control Medicine, Graduate School of Medicine, Kyoto University, Kyoto, Japan

Tel. +8175-751-3852

*Prof. Michiaki Mishima*

*Dr. Yuichi Chihara*

*Dr. Yuka Harada*

*Dr. Kimihiko Murase*

*Dr. Masanori Azuma*

*Dr. Yoshiro Toyama*

The Department of Respiratory Medicine, Graduate School of Medicine, Kyoto University, Kyoto, Japan

Tel. +8175-751-3852

***12. References***

1. Browning JD, Szczepaniak LS, Dobbins R*, et al*. Prevalence of hepatic steatosis in an urban population in the United States: Impact of ethnicity. *Hepatology.* 2004;40:1387-1395.
2. Bellentani S, Saccoccio G, Masutti F*, et al*. Prevalence of and risk factors for hepatic steatosis in northern Italy. *Ann Intern Med.* 2000;132:112-117.
3. Jimba S, Nakagami T, Takahashi M*, et al*. Prevalence of non-alcoholic fatty liver disease and its association with impaired glucose metabolism in Japanese adults. *Diabetic Med.* 2005;22:1141-1145.
4. Kojima S-, Watanabe N, Numata M*, et al*. Increase in the prevalence of fatty liver in Japan over the past 12 years: Analysis of clinical background. *J Gastroenterol.* 2003;38:954-961.
5. Ekstedt M, Franzén LE, Mathiesen UL*, et al*. Long-term follow-up of patients with NAFLD and elevated liver enzymes. *Hepatology.* 2006;44:865-873.
6. Sanyal AJ, Banas C, Sargeant C*, et al*. Similarities and differences in outcomes of cirrhosis due to nonalcoholic steatohepatitis and hepatitis C. *Hepatology.* 2006;43:682-689.
7. Dixon JB, Bhathal PS, O'Brien PE, *et al.* Nonalcoholic fatty liver disease: Predictors of nonalcoholic steatohepatitis and liver fibrosis in the severely obese. *Gastroenterology.* 2001;121:91-100.
8. Eguchi Y, Eguchi T, Mizuta T, *et al.* Visceral fat accumulation and insulin resistance are important factors in nonalcoholic fatty liver disease. *J.Gastroenterol.* 2006;41:462-469.
9. Speliotes EK, Massaro JM, Hoffmann U, *et al.* Fatty liver is associated with dyslipidemia and dysglycemia independent of visceral fat: The Framingham heart study. *Hepatology.* 2010;51:1979-1987.
10. Iwasaki M, Takada Y, Hayashi M*, et al*. Noninvasive evaluation of graft steatosis in living donor liver transplantation. *Transplantation.* 2004;78:1501-1505.
11. Park SH, Kim PN, Kim KW*, et al*. Macrovesicular hepatic steatosis in living liver donors: Use of CT for quantitative and qualitative assessment. *Radiology.* 2006;239:105-112.
12. Sang WL, Seong HP, Kyoung WK*, et al*. Unenhanced CT for assessment of macrovesicular hepatic steatosis in living liver donors: Comparison of visual grading with liver attenuation index. *Radiology.* 2007;244:479-485.
13. Lee SS, Park SH, Kim HJ*, et al*. Non-invasive assessment of hepatic steatosis: Prospective comparison of the accuracy of imaging examinations. *J Hepatol.* 2010;52:579-585.
14. Dixon JB, Bhathal PS, O'Brien PE, *et al.* Nonalcoholic fatty liver disease: Predictors of nonalcoholic steatohepatitis and liver fibrosis in the severely obese. *Gastroenterology.* 2001;121:91-100.
15. Eguchi Y, Eguchi T, Mizuta T, *et al.* Visceral fat accumulation and insulin resistance are important factors in nonalcoholic fatty liver disease. *J.Gastroenterol.* 2006;41:462-469.
16. Speliotes EK, Massaro JM, Hoffmann U, *et al.* Fatty liver is associated with dyslipidemia and dysglycemia independent of visceral fat: The Framingham heart study. *Hepatology.* 2010;51:1979-1987.
17. Chin K, Shimizu K, Nakamura T, *et al.* Changes in intra-abdominal visceral fat and serum leptin levels in patients with obstructive sleep apnea syndrome following nasal continuous positive airway pressure therapy. *Circulation.* 1999;100:706-712.
18. Ip MSM, Lam B, Ng MMT, *et al.* Obstructive sleep apnea is independently associated with insulin resistance. *Am J Respir Crit Care Med.* 2002;165:670-676.
19. Punjabi NM, Sorkin JD, Katzel LI, *et al.* Sleep-disordered breathing and insulin resistance in middle-aged and overweight men. *Am J Respir Crit Care Med.* 2002:165;677-682.
20. Jouët P, Sabaté J-, Maillard D*, et al*. Relationship between obstructive sleep apnea and liver abnormalities in morbidly obese patients: A prospective study. *Obesity Surg.* 2007;17:478-485.
21. Norman D, Bardwell WA, Arosemena F*, et al*. Serum aminotransferase levels are associated with markers of hypoxia in patients with obstructive sleep apnea. *Sleep.* 2008;31:121-126.
22. Tanné F, Gagnadoux F, Chazouillères O*, et al*. Chronic liver injury during obstructive sleep apnea. *Hepatology.* 2005;41:1290-1296.
23. Li J, Grigoryev DN, Ye SQ*, et al*. Chronic intermittent hypoxia upregulates genes of lipid biosynthesis in obese mice. *J Appl Physiol.* 2005;99:1643-1648.
24. Harsch IA, Schahin SP, Radespiel-Tröger M, et al. Continuous Positive Airway Pressure Treatment Rapidly Improves Insulin Sensitivity in Patients with Obstructive Sleep Apnea Syndrome. *Am J Respir Crit Care Med.* 2004;169:156-162.
25. Chin K, Nakamura T, Takahashi K*, et al*. Effects of obstructive sleep apnea syndrome on serum aminotransferase levels in obese patients. *Am J Med.* 2003;114:370-376.
26. Shpirer I, Copel L, Broide E*, et al*. Continuous positive airway pressure improves sleep apnea associated fatty liver. *Lung.* 2010;188:301-307.
27. Daltro C, Cotrim HP, Alves E*, et al*. Nonalcoholic fatty liver disease associated with obstructive sleep apnea: Just a coincidence? *Obesity Surg.* 2010;20:1536-1543.
