# Supplementary material for: Impact of Obstructive Sleep Apnea on Liver Fat Accumulation According to Sex and Visceral Obesity
Source: PLoS One. 2015 Jun 15;10(6):e0129513. doi: 10.1371/journal.pone.0129513 (PMC4468199; doi:10.1371/journal.pone.0129513)
Supplement: S2 File — (DOC) [file pone.0129513.s003.doc]

**研究実施計画書**

**【研究の背景】**

相応の飲酒歴がないにも関わらず肝細胞の大滴性脂肪沈着を呈する疾患は非アルコール性脂肪肝疾患と呼ばれ、現在では成人の3～4人に1人が罹患しているとされる[1-4]。従来は非進行性で良性と考えられていた単純性脂肪肝も、脂肪肝炎に進行して肝硬変や肝細胞癌の原因になりえることが分かっており、臨床上重要な問題となっている[5,6]。また近年、脂肪肝の進行に関し内臓脂肪量、インスリン抵抗性の関与が指摘されている[7-9]。脂肪肝診断のGold standardは肝生検による病理組織診断であるが、検査に伴う侵襲や高い罹患率のために全例で実施するのは困難であり、より簡便な診断法が模索されている。その中でも肝CT値は脂肪肝の診断に有用であることが示されている[10-13]。

　閉塞性睡眠時無呼吸（Obstructive Sleep Apnea: OSA）は、睡眠中に間欠的低酸素血症を繰り返し引き起こして交感神経興奮や酸化ストレスの原因となり、心脳血管障害、高血圧、インスリン抵抗性増強などさまざまな合併症の危険因子となることが分かっている。近年、OSAと内臓脂肪量、インスリン抵抗性の関係が注目されており[14-16]、また、夜間の低酸素血症が肝酵素値異常[17,18]や脂肪肝炎[19]の危険因子となることが示唆されており、動物実験でも間欠的低酸素の暴露によって肝の脂肪含量が増加することが示されている[20]。またCPAP療法によるOSA患者の内臓脂肪量、インスリン抵抗性、肝酵素値改善が報告され[14,21,22]、小規模ではあるが肝CT値を用いた研究でCPAPによる脂肪肝改善が示唆されている[23]。一方、OSAと内臓脂肪量、脂肪肝の関連については否定的な報告もある[17,24]。

このようにOSAが内臓脂肪量、脂肪肝に与える影響や、OSAをCPAPで治療することで内臓脂肪量、脂肪肝にどの程度の改善が認められるかについては、よく分かっていない。当院において、メタボリックシンドロームや内臓脂肪型肥満の診断目的で実施されるCTには肝臓も撮像されていることが多く、これを用いて、内臓脂肪量の変化だけでなく撮像された範囲内での脂肪肝も評価することを目的として、本研究を実施することにした。

**【研究の目的・意義】**

　OSAが疑われて当院でポリソムノグラフィ（Polysomnography: PSG）を実施され、またメタボリックシンドロームまたは内臓脂肪型肥満症の診断のために京大病院の規定のCTで腹部内臓脂肪を測定された患者のうち、肝臓のCT値が利用可能な患者について、OSAの重症度指標と同CT画像における肝CT値の関連を、内臓脂肪量やその他の血清学的検査とともに検討する。さらにOSAのため導入されたCPAP療法を6ヶ月以上継続している患者が、一度目のCTでメタボリックシンドローム、内臓脂肪型肥満、脂肪肝を合併している場合、治療効果や病状の評価のために内臓脂肪CTを実施し、これを用いて治療前後の内臓脂肪、肝CT値の変化を検討し、同時に血清学的検査の変化についても検討する。

**1. 研究デザインの種類**

横断研究、コホート研究

**2. 研究期間**

1）対象者登録期間

倫理委員会承認日より2年間。

2）対象者追跡期間

追跡調査は、OSAに対してCPAP治療導入となり、当院に通院中の患者を対象とする。

**3. 対象者の選択**

1）選択の場（施設および機会、その合理性）

京都大学医学部附属病院

2）包含基準および除外基準

包含基準

いびきや睡眠時の呼吸障害などOSAを疑わせる訴えがあり、京都大学医学部附属病院においてPSG検査を実施された成人（20歳以上）のうち、メタボリックシンドロームまたは内臓脂肪型肥満症の診断のために腹部CTを実施された症例を対象とする。さらにCPAP療法における追跡評価に関しては、OSAと診断されCPAP療法を導入された患者のうち、6ヶ月以上CPAP療法を継続し当院に通院中で、初回の内臓脂肪量検査にてメタボリックシンドローム、内臓脂肪型肥満、脂肪肝が指摘された患者のうち、内臓脂肪CT検査に同意した症例を対象とする。

除外基準

・診断PSG施行時にすでに睡眠呼吸障害の治療を受けているもの。

・肝疾患や肝炎ウイルス感染が明らかな患者、うっ血や重篤な合併症を有する患者。

・他疾患のために薬剤性脂肪肝の原因となる薬剤（アミオダロン、メソトレキセートなど）の投与を受けている患者。

・研究参加について自由意志による決定が困難と思われる症例。

3）標本規模

・登録期間中に、診断のために当院でPSG検査を実施した患者のうち、包含基準を満たした患者を評価対象とする。

**4. 介入や追跡の方法**

PSGの結果でOSAの重症度を評価するが、肝CT値の評価は当院の内臓脂肪測定用のCT検査で撮像範囲に含まれた肝について行う。PSGでCPAP療法の適応と判断され、同治療導入後も当院に通院し6ヶ月以上治療を継続中の症例のうち、一度目の検査でメタボリックシンドローム、内臓脂肪型肥満、脂肪肝が明らかになった患者において同意を得て内臓脂肪計測用の腹部CTを実施し、撮像範囲に含まれた肝についてはCT値を測定するとともに、身体測定、生化学検査、各種液性因子の測定を行う。CPAPによる治療状況は、使用機器に付属の記録媒体より得られるデータから評価する。

**5. 検討項目の測定**

測定機関：京都大学医学部附属病院

1) 診断PSG実施時

① 患者背景

・年齢、性別、身長、体重、BMI、腹囲、頚部周囲径、血圧

・既往歴、喫煙歴、飲酒量、その他の治療内容

② QOL、睡眠アンケート

・Epworth Sleepiness Scale (ESS): 眠気の評価

・Medical Outcomes Study 36-Item Short From Health Survey (SF-36): 一般的健康状態の評価

・Pittsburgh Sleep Quality Index (PSQI): 睡眠の質の評価

・Morning Eveningness Questionnaire: 朝型か夜型かの評価

・Calgary Sleep Apnea Quality of Life Index (SAQLI): 疾患特異的な健康状態の評価

・British Medical Research Council Dyspnea Scale (MRC): 呼吸困難の評価

・Hospital Anxiety and Depression Scale (HADS): 不安抑うつの評価

・Physical Activity: 日常活動の評価

③ PSG

④ 腹部単純CT

・20歳以上の男性または40歳以上の女性でメタボリックシンドローム、内臓脂肪型肥満が疑われ、かつ本人の希望または同意が得られた場合、内臓脂肪量評価のために保険診療の範囲で当院にて実施される通常の腹部内臓脂肪量測定用のCTを施行する。

・⑤ 血液検査（空腹時）、尿検査

・血球数、肝機能、腎機能、電解質、糖代謝、脂質代謝、炎症マーカー、各種ホルモンおよび液性因子、など

・動脈血液ガス

⑥ 心電図・呼吸機能検査

※これらのうち④以外は全て、OSA診断のためのPSG入院時に全患者で実施されるルーチン検査である。

2) CPAP導入後（6ヶ月以上）

① 患者背景（年齢、身長、体重、BMI、腹囲、頚部周囲径、血圧、服薬内容の変更）

② CPAPカード解析（AHI、コンプライアンス）

③ 初回検査でメタボリックシンドローム、内臓脂肪型肥満、脂肪肝の評価を受けた患者で同意が得られれば、内臓脂肪測定用のCTを行う。

④ 血液検査（肝機能、脂質代謝など）

**6. 介入や測定によってあらたに加わる侵襲と予想される有害事象**

検査については採血に伴う侵襲と腹部単純CT実施に伴う被曝が考えられるが、被験者はメタボリックシンドローム、内臓脂肪型肥満が疑われた患者で、京大病院内における通常の内臓脂肪測定用のCT画像より肝臓のCT値が測定できる患者のみについて同意のもとで行うので、本研究により新たな被曝が生じることはない。また一度目のCT検査にて異常を認めた群に対してはCPAP治療のみならず減量指導なども行っており、二度目のCTは、これらの患者を対象として治療効果を判定し今後の治療方針を検討することを目的として、同意のもとで撮影される。従って異常を認めなかった者は対象とならず、不要な被曝は生じない。介入にあたるCPAP療法は、研究とは関係なく一般診療において同療法の適応となった患者に導入されたものであり、研究に伴い新たに加わる侵襲や有害事象はないと考えられる。

**7. 疫学研究としての解析の概要**

・OSAの重症度指標（Apnea Hypopnea Index, AHI、SpO2 90%以下時間、睡眠中平均SpO2、睡眠中最低SpO2、覚醒指数など）と脂肪肝のCT指標（肝CT値、肝脾CT値差）が、その他の関連因子から独立して関連しているか、多変量解析で検討する。

・メタボリックシンドローム、内臓脂肪型肥満を合併するOSA患者について、CPAP治療によるOSA重症度指標の改善と内臓脂肪および肝CT値の変化が、その他の関連因子の変化とは独立して認められるか、多変量解析で検討する。

**8. 個人情報（個人の同定が可能もの）の保護**

本研究においては被験者の連結不可能匿名化を行い、プライバシーの保護に務める。匿名化に用いる研究番号と患者IDの対応表、および匿名化されたデータベースへのアクセスは、パスワードまたは指紋認証によるセキュリティー管理下におかれ、本研究実施責任者および分担研究者以外のアクセスは不可能となる。解析終了後は適切に廃棄する。

**9. 対象者への説明・同意と倫理面への配慮**

本検討は「ヘルシンキ宣言」と「疫学研究に関する倫理指針」、「臨床研究に関する倫理指針の施行等について」にもとづき実施する。対象者本人に、担当医師または臨床担当者から、本研究への協力の同意および人権保護、プライバシー保護、調査研究の内容について説明書および口頭で説明する。文書により自由意志による同意を得るものとする（別紙）。また、その同意に関する記録を1通残すものとする。本人の理解と同意を得ることが困難と思われる症例については、本研究の対象としない。本研究への参加は自由意志で決めることができ、不参加であっても不利益が生じないこと、いつでも参加の取り消しができることを説明する。

**10. 費用負担および謝礼**

1）検査・薬剤等の費用負担

検査および導入されたCPAPは病状評価と治療のため必要であり一般診療の範囲内であるので、全て保険診療で実施する。

2）研究参加の謝礼

本研究参加に対する謝礼は設けない。

**11. 研究組織**

・主任研究者

京都大学大学院医学研究科呼吸管理睡眠制御学　　　　　　　　　　　　　教授　　　　陳　和夫

連絡先：〒606-8507　京都府京都市左京区聖護院川原町54

電話番号：075-751-3852

・共同研究者

京都大学医学部医学研究科呼吸器内科学　　　　　　　　　　　　　　　　教授　　　三嶋　理晃

・担当医師

京都大学大学院医学研究科呼吸管理睡眠制御学　　　　　　　　　　　　　准教授　　　小賀　徹

同上　　　　　　　　　　　　　　　　　　　　　　　　　　　　　　　助教　　　人見　健文

同上　　　　　　　　　　　　　　　　　　　　　　　　　　　　　　　助教　　　　吉村　力

京都大学大学院医学研究科呼吸器内科学　　　　　　　　　　　　　　　　研究生　　茆原　雄一

同上　　　　　　　　　　　　　　　　　　　　　　　　　　　　　　　大学院生　原田　有香

同上　　　　　　　　　　　　　　　　　　　　　　　　　　　　　　　大学院生　村瀬　公彦

同上　　　　　　　　　　　　　　　　　　　　　　　　　　　　　　　大学院生　　東　正徳

同上　　　　　　　　　　　　　　　　　　　　　　　　　　　　　　　大学院生　外山　善朗

**12. 参考文献**

1. Browning JD, Szczepaniak LS, Dobbins R*, et al*. Prevalence of hepatic steatosis in an urban population in the United States: Impact of ethnicity. *Hepatology.* 2004;40:1387-1395.
2. Bellentani S, Saccoccio G, Masutti F*, et al*. Prevalence of and risk factors for hepatic steatosis in northern Italy. *Ann Intern Med.* 2000;132:112-117.
3. Jimba S, Nakagami T, Takahashi M*, et al*. Prevalence of non-alcoholic fatty liver disease and its association with impaired glucose metabolism in Japanese adults. *Diabetic Med.* 2005;22:1141-1145.
4. Kojima S-, Watanabe N, Numata M*, et al*. Increase in the prevalence of fatty liver in Japan over the past 12 years: Analysis of clinical background. *J Gastroenterol.* 2003;38:954-961.
5. Ekstedt M, Franzén LE, Mathiesen UL*, et al*. Long-term follow-up of patients with NAFLD and elevated liver enzymes. *Hepatology.* 2006;44:865-873.
6. Sanyal AJ, Banas C, Sargeant C*, et al*. Similarities and differences in outcomes of cirrhosis due to nonalcoholic steatohepatitis and hepatitis C. *Hepatology.* 2006;43:682-689.
7. Dixon JB, Bhathal PS, O'Brien PE, *et al.* Nonalcoholic fatty liver disease: Predictors of nonalcoholic steatohepatitis and liver fibrosis in the severely obese. *Gastroenterology.* 2001;121:91-100.
8. Eguchi Y, Eguchi T, Mizuta T, *et al.* Visceral fat accumulation and insulin resistance are important factors in nonalcoholic fatty liver disease. *J.Gastroenterol.* 2006;41:462-469.
9. Speliotes EK, Massaro JM, Hoffmann U, *et al.* Fatty liver is associated with dyslipidemia and dysglycemia independent of visceral fat: The Framingham heart study. *Hepatology.* 2010;51:1979-1987.
10. Iwasaki M, Takada Y, Hayashi M*, et al*. Noninvasive evaluation of graft steatosis in living donor liver transplantation. *Transplantation.* 2004;78:1501-1505.
11. Park SH, Kim PN, Kim KW*, et al*. Macrovesicular hepatic steatosis in living liver donors: Use of CT for quantitative and qualitative assessment. *Radiology.* 2006;239:105-112.
12. Sang WL, Seong HP, Kyoung WK*, et al*. Unenhanced CT for assessment of macrovesicular hepatic steatosis in living liver donors: Comparison of visual grading with liver attenuation index. *Radiology.* 2007;244:479-485.
13. Lee SS, Park SH, Kim HJ*, et al*. Non-invasive assessment of hepatic steatosis: Prospective comparison of the accuracy of imaging examinations. *J Hepatol.* 2010;52:579-585.
14. Dixon JB, Bhathal PS, O'Brien PE, *et al.* Nonalcoholic fatty liver disease: Predictors of nonalcoholic steatohepatitis and liver fibrosis in the severely obese. *Gastroenterology.* 2001;121:91-100.
15. Eguchi Y, Eguchi T, Mizuta T, *et al.* Visceral fat accumulation and insulin resistance are important factors in nonalcoholic fatty liver disease. *J.Gastroenterol.* 2006;41:462-469.
16. Speliotes EK, Massaro JM, Hoffmann U, *et al.* Fatty liver is associated with dyslipidemia and dysglycemia independent of visceral fat: The Framingham heart study. *Hepatology.* 2010;51:1979-1987.
17. Chin K, Shimizu K, Nakamura T, *et al.* Changes in intra-abdominal visceral fat and serum leptin levels in patients with obstructive sleep apnea syndrome following nasal continuous positive airway pressure therapy. *Circulation.* 1999;100:706-712.
18. Ip MSM, Lam B, Ng MMT, *et al.* Obstructive sleep apnea is independently associated with insulin resistance. *Am J Respir Crit Care Med.* 2002;165:670-676.
19. Punjabi NM, Sorkin JD, Katzel LI, *et al.* Sleep-disordered breathing and insulin resistance in middle-aged and overweight men. *Am J Respir Crit Care Med.* 2002:165;677-682.
20. Jouët P, Sabaté J-, Maillard D*, et al*. Relationship between obstructive sleep apnea and liver abnormalities in morbidly obese patients: A prospective study. *Obesity Surg.* 2007;17:478-485.
21. Norman D, Bardwell WA, Arosemena F*, et al*. Serum aminotransferase levels are associated with markers of hypoxia in patients with obstructive sleep apnea. *Sleep.* 2008;31:121-126.
22. Tanné F, Gagnadoux F, Chazouillères O*, et al*. Chronic liver injury during obstructive sleep apnea. *Hepatology.* 2005;41:1290-1296.
23. Li J, Grigoryev DN, Ye SQ*, et al*. Chronic intermittent hypoxia upregulates genes of lipid biosynthesis in obese mice. *J Appl Physiol.* 2005;99:1643-1648.
24. Harsch IA, Schahin SP, Radespiel-Tröger M, et al. Continuous Positive Airway Pressure Treatment Rapidly Improves Insulin Sensitivity in Patients with Obstructive Sleep Apnea Syndrome. *Am J Respir Crit Care Med.* 2004;169:156-162.
25. Chin K, Nakamura T, Takahashi K*, et al*. Effects of obstructive sleep apnea syndrome on serum aminotransferase levels in obese patients. *Am J Med.* 2003;114:370-376.
26. Shpirer I, Copel L, Broide E*, et al*. Continuous positive airway pressure improves sleep apnea associated fatty liver. *Lung.* 2010;188:301-307.
27. Daltro C, Cotrim HP, Alves E*, et al*. Nonalcoholic fatty liver disease associated with obstructive sleep apnea: Just a coincidence? *Obesity Surg.* 2010;20:1536-1543.
