# Supplementary material for: Impact of Obstructive Sleep Apnea on Liver Fat Accumulation According to Sex and Visceral Obesity
Source: PLoS One. 2015 Jun 15;10(6):e0129513. doi: 10.1371/journal.pone.0129513 (PMC4468199; doi:10.1371/journal.pone.0129513)
Supplement: S1 Table — (DOC) [file pone.0129513.s004.doc]

**S1 Table. Stepwise multiple regression models for CT_LFA_ in females with and without VO_100_**

|  | Females with VO_100_ (n=32) | | |  | Females without VO_100_ (n=30) | | |
| --- | --- | --- | --- | --- | --- | --- | --- |
| *Variables* | Β | R^2^, % | *P* value |  | β | R^2^, % | *P* value |
| Age | 0.43 | 24.5 | 0.004 |  | − | − | − |
| Neck circumference | − | − | − |  | -0.76 | 56.7 | <0.001 |
| VFA | -0.33 | 13.2 | 0.024 |  | − | − | − |
| Triglycerides | -0.35 | 13.6 | 0.014 |  | − | − | − |
| Fasting blood glucose | − | − | − |  | -0.41 | 15.2 | <0.001 |
| *Cumulative R^2^* | … | 51.4 | … |  | … | 71.9 | … |
|  |  |  |  |  |  |  |  |

Variables entered into the stepwise regression analyses were selected from age, BMI, neck circumference, waist circumference, systolic and diastolic blood pressures, alcohol intake, use of lipid-lowering agents, current smoking, VFA, SFA, AHI, 4%ODI, %T<90, lowest SpO_2_, arousal index, REM sleep, AHI during REM, supine sleep time, AHI in supine position, Epworth sleepiness scale score, CRP, triglycerides, HDL-cholesterol, LDL-cholesterol, fasting plasma glucose, and HOMA-IR when yielding a P-value <0.10 by univariate analysis; only variables left in one or more of the models are shown in this table. Minus sign means the variable was not selected through univariate or stepwise selection procedures. CT_LFA_ is negatively correlated with liver fat accumulation.

*Abbreviations:* CT_LFA_, CT values for liver; VO_100_, visceral obesity (VFA ≥100 cm^2^); β = standard regression coefficient; R^2^ = coefficient of determination; VFA, visceral fat accumulation.
