# Supplementary material for: Impact of Obstructive Sleep Apnea on Liver Fat Accumulation According to Sex and Visceral Obesity
Source: PLoS One. 2015 Jun 15;10(6):e0129513. doi: 10.1371/journal.pone.0129513 (PMC4468199; doi:10.1371/journal.pone.0129513)
Supplement: S2 Table — (DOC) [file pone.0129513.s005.doc]

**S2 Table. Stepwise multiple regression models for CT_LFA_ in males with and without obesity (BMI ≥ 25 kg/m^2^**).

|  | Males with obesity (n=110) | | |  | Males without obesity (n=78) | | |
| --- | --- | --- | --- | --- | --- | --- | --- |
| *Variables* | Β | R^2^, % | *P* value |  | β | R^2^, % | *P* value |
| Age | 0.28 | 10.0 | 0.004 |  | − | − | − |
| BMI | -0.24 | 10.4 | 0.013 |  | -0.26 | 9.5 | 0.014 |
| VFA | -0.25 | 6.9 | 0.007 |  | − | − | − |
| Triglycerides | -0.18 | 8.3 | 0.031 |  | − | − | − |
| HDL-cholesterol | − | − | − |  | 0.26 | 8.7 | 0.011 |
| HOMA-IR | − | − | − |  | -0.23 | 7.2 | 0.029 |
| *Cumulative R^2^* | … | 32.6 | … |  | … | 25.4 | … |

Variables entered into the stepwise regression analyses were selected from age, BMI, neck circumference, waist circumference, systolic and diastolic blood pressures, alcohol intake, use of lipid-lowering agents, current smoking, VFA, SFA, AHI, 4%ODI, %T<90, lowest SpO_2_, arousal index, REM sleep, AHI during REM, supine sleep time, AHI in supine position, Epworth sleepiness scale score, CRP, triglycerides, HDL-cholesterol, LDL-cholesterol, fasting plasma glucose, and HOMA-IR when yielding a P-value <0.10 by univariate analysis; only variables left in one or more of the models are shown in this table. Minus sign means the variable was not selected through univariate or stepwise selection procedures. CT_LFA_ is negatively correlated with liver fat accumulation.

*Abbreviations:* CT_LFA_, CT values for liver; β = standard regression coefficient; R^2^ = coefficient of determination; BMI, body mass index; VFA, visceral fat accumulation; HOMA-IR, homeostasis model assessment index of insulin resistance.
